# Comparison of the *Legionella pneumophila* population structure as determined by sequence-based typing and whole genome sequencing

**DOI:** 10.1186/1471-2180-13-302

**Published:** 2013-12-24

**Authors:** Anthony P Underwood, Garan Jones, Massimo Mentasti, Norman K Fry, Timothy G Harrison

**Affiliations:** 1 Bioinformatics Unit, Microbiology Services (Colindale), Public Health England, 61 Colindale Avenue, London, NW9 5EQ, UK; 2 Respiratory and Vaccine Preventable Bacteria Reference Unit, Microbiology Services (Colindale), Public Health England, 61 Colindale Avenue, London, NW9 5EQ, UK; 3 Centre for Bioinformatics, Biosciences, College of Life and Environmental Sciences, Geoffrey Pope, University of Exeter, Stocker Road, Exeter, EX4 4QD, UK

**Keywords:** *Legionella pneumophila*, Sequence-based typing, Whole genome sequencing, Clustering, Population structure, Recombination

## Abstract

**Background:**

*Legionella pneumophila* is an opportunistic pathogen of humans where the source of infection is usually from contaminated man-made water systems. When an outbreak of Legionnaires’ disease caused by *L. pneumophila* occurs, it is necessary to discover the source of infection. A seven allele sequence-based typing scheme (SBT) has been very successful in providing the means to attribute outbreaks of *L. pneumophila* to a particular source or sources. Particular sequence types described by this scheme are known to exhibit specific phenotypes. For instance some types are seen often in clinical cases but are rarely isolated from the environment and vice versa. Of those causing human disease some types are thought to be more likely to cause more severe disease. It is possible that the genetic basis for these differences are vertically inherited and associated with particular genetic lineages within the population. In order to provide a framework within which to test this hypothesis and others relating to the population biology of *L. pneumophila,* a set of genomes covering the known diversity of the organism is required.

**Results:**

Firstly, this study describes a means to group *L. pneumophila* strains into pragmatic clusters, using a methodology that takes into consideration the genetic forces operating on the population. These clusters can be used as a standardised nomenclature, so those wishing to describe a group of strains can do so. Secondly, the clusters generated from the first part of the study were used to select strains rationally for whole genome sequencing (WGS). The data generated was used to compare phylogenies derived from SBT and WGS. In general the SBT sequence type (ST) accurately reflects the whole genome-based genotype. Where there are exceptions and recombination has resulted in the ST no longer reflecting the genetic lineage described by the whole genome sequence, the clustering technique employed detects these sequence types as being admixed, indicating their mixed inheritance.

**Conclusions:**

We conclude that SBT is usually a good proxy for the genetic lineage described by the whole genome, and therefore utility of SBT is still suitable until the technology and economics of high throughput sequencing reach the point where routine WGS of *L. pneumophila* isolates for outbreak investigation is feasible.

## Background

Legionellosis is acquired by inhalation or aspiration of *Legionella* spp. from a contaminated environmental source. Thus, when a case of legionellosis is recognized others may become infected from the same source if appropriate control measures are not taken to reduce the risk of further transmission. The source of the outbreak or incident can be determined by epidemiological investigation together with characterization of legionellae isolated from patients and putative environmental sources [1,2].

As the vast majority of cases of legionellosis are caused by *Legionella pneumophila*, and this species is very common in the environment, discriminatory typing methods are needed to differentiate between isolates if a convincing epidemiological link between patient and source is to be established. Consequently a large number of molecular methods have been investigated for epidemiological typing purposes and one of these, devised by members of the European Working Group for Legionella Infections (EWGLI) and termed sequence-based typing (SBT), has become established internationally as the typing method of choice [3,4]. This method is a variant of the classic multi-locus sequence typing (MLST) schemes used to identify bacterial lineages, the utility of which has been previously described [5].

The availability of a substantial quantity of international SBT typing data has led to the recognition that the majority of legionellosis is caused by a relatively small subset of all strains recovered from the environment [6,7]. This poses the question of whether some clonal lineages have characteristics that make them more likely to cause human infection than others that are more, or equally, prevalent in the environment [6]. Requirements to answer this question are; a means to subdivide the *L. pneumophila* population into clusters which are genetically similar so that we can describe the shared phenotypes of these clusters, and knowledge of the frequency of horizontal gene transfer (HGT) and recombination. This latter is crucial since these molecular events may result in the rapid development of novel phenotypes previously unseen in a clonal lineage and high levels of recombination may make clustering of organisms into related groups problematic [8].

Early studies using electrophoretic analysis of protein polymorphism (multi locus enzyme electrophoresis, MLEE) described 62 electrophoretic types and concluded that *L. pneumophila* was clonal in nature [9]. More recently a study examining four genes in the dot/icm complex [10] demonstrated clear evidence of intraspecific genetic exchange in *L. pneumophila*. Whilst initial studies using SBT data [11,12] supported evidence for the clonal nature of *L.pneumophila*, it was acknowledged that intergenic recombination events could not be ruled out. Subsequent work analysing intragenic recombination in the six SBT loci and additional non-coding loci concluded that recombination was frequent in *Legionella* spp. [13,14]*.* Research which concentrated on the genes involved in the type II secretion system concluded that intragenic recombination was a rare event but there was good evidence for intergenic recombination [15]. Recent analysis that looked for recombination throughout the whole genome revealed significant levels of HGT both within the species *L. pneumophila* and from other Gamma-Proteobacteria especially those, that like legionellae, are associated with amoebae [16]. A comprehensive review of the current knowledge about the population genetics, phylogenetics and genome of *L. pneumophila* concluded that recombination is playing a role in diversifying the species but this may have been more significant in the past than is seen with the current population of the species [17].

The EWGLI SBT database has now grown significantly since the work described in earlier publications with the addition of a seventh allele (*neuA*) and the designation of Sequence Types (STs) [18]. The database contained 838 distinct sequence types at the time of this study and these were derived from strains isolated from worldwide locations in contrast to other studies that used more localised samples sets.

Therefore, in light of this large increase in novel STs, the aims of this study were;

1) To evaluate this global dataset and assess the relative contribution of recombination mediated by HGT and mutation to genome evolution.

2) To derive a method to cluster strains of similar genotype based on the type of population structure found in the first part of this study. This would provide a set of pragmatic groups that could be labelled and referred to using a common terminology within the *Legionella* scientific community.

3) To sequence the genomes of isolates representative of these major clusters within the population and provide an overview of the population structure. This would enable comparison of the genetic types determined by SBT with that derived by examining the diversity within the whole genome.

4) The ultimate aim was to provide a set of sequenced strains, which adequately represent the *L. pneumophila* pan genome. This will enable further studies where strains within a cluster are investigated in more detail, and allow testing of the hypothesis that clusters of strains are likely to share a common lineage and therefore some phenotypic similarities.

## Results and Discussion

### Sequence Based Typing analysis: Recombination Tests

Choice of the best algorithm with which to cluster the sequence types of *L. pneumophila* will be informed by the population structure of the species, which will in turn be influenced by the relative contributions of recombination and mutation to sequence evolution. Therefore the frequencies of intergenic and intragenic recombination in *L. pneumophila* were investigated and compared to those for *Staphlococcus aureus* (representing a comparatively clonal species), *Streptococcus pneumoniae* (representing an intermediate species) and *Neisseria meningitidis* (representating a panmictic species).

Intergenic recombination is an event by which one allele of a locus can be replaced by another by horizontal gene transfer. The standardised index of association ( $I_{S}^{A}$) is a commonly used measure of intergenic recombination. Another measure of recombination over more than just one locus is the r/m ratio. This is the ratio of probabilities that a base change occurs by recombination or mutation.

The results for these two tests (Table 1) are in agreement for each of the four species apart from *N. meningitidis* where the value of $I_{S}^{A}$ is anomalous being higher than that for *S. pneumoniae*. There has been the suggestion that sample bias may cause dramatic effects on the value for $I_{S}^{A}$ giving a distorted value. This effect may be diminished by including just a single example of each sequence type but the removal of many isolates can reduce the ability to estimate the extent of recombination from linkage disequilibrium [19]. Our analysis included just one example of each ST, but the $I_{S}^{A}$ value for *N. meningitidis* is still higher than would be expected. As noted by others [20,21] a high $I_{S}^{A}$ value does not necessarily infer clonality since linkage disequilibrium can still be observed in species that are highly recombining due to population structuring as observed in *Helicobacter pylori* for example [22]. Therefore the high value of $I_{S}^{A}$ for *N. meningitidis* may indicate a highly structured population such that the epidemic epidemiology leads to a superficially clonal population [20]. Based on these results overall *L. pneumophila* has intermediate levels of recombination between those of *S. aureus* and *N. meningitidis*. The value of $I_{S}^{A}$ indicates a population that tends towards being clonal, although again this may be due to a very structured population.

**Table 1:** Values of the standardised index of association and recombination to mutation ratio

	**Standardised Index of Association (**$I_{S}^{A}$**)**	**Recombination to mutation ratio (r/m)**
*Staphylococcus aureus* (Clonal)	0.193	1.6
*Streptococcus pneumoniae* (Intermediate)	0.044	9.3
*Neisseria menigitidis* (Panmictic)	0.116	32.5
*Legionella pneumophila*	0.153	16.8

**Figure 1:**
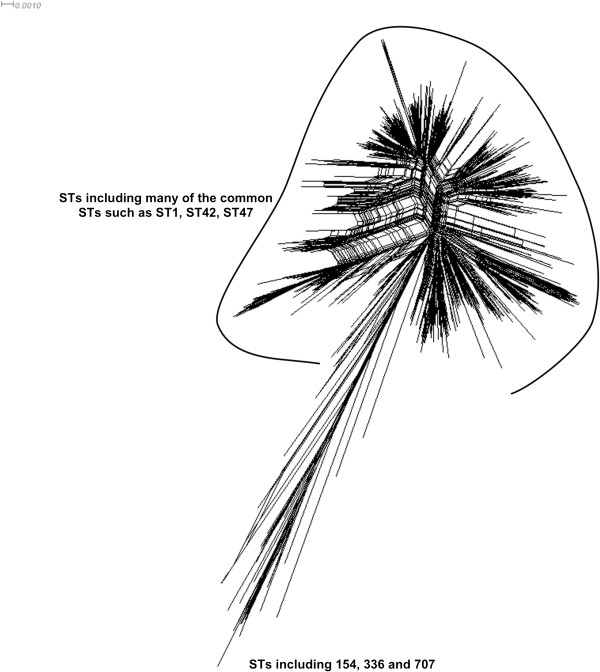
A reticulate tree generated by the Neighbor-net algorithm of SplitsTree4 using the concatenated sequences of the seven SBT loci as input data.

Based on the sequences from SBT a reticulate network tree was drawn using the Neighbor-net algorithm of SplitsTree. Reticulate networks attempt to provide a more ‘explicit’ representation of evolutionary history than traditional phylogenetic trees such as phylograms. They are often depicted as a phylogenetic tree with additional edges. The internal nodes in this network represent ancestral species, and nodes with more than two parents correspond to ‘reticulate’ events such as recombination: the more splits in the branches seen in the resulting tree the more recombination or HGT is likely to have taken place. The SplitsTree computed from the *L. pneumophila* data (Figure 1) gives strong evidence for significant recombination between a subset of the lineages present within the tree and yields a highly significant phi test (p = 0.0).

The results of the tests for examining intragenic recombination (recombination within the sequence of a gene) are summarised in Table 2. For each test the number of loci that were positive for recombination is recorded. For RDP at least two of the individual tests in the suite had to be positive in order for the locus to be scored positive overall.

**Table 2:** Number of loci positive for recombination by the Sawyer’s run test and RDP suite

	**Sawyer’s run test**	**RDP tests**
*Staphylococcus aureus* (Clonal)	0 loci	1 locus
*Streptococcus pneumoniae* (Intermediate)	3 loci	4 loci
*Neisseria menigitidis* (Panmictic)	7 loci	6 loci
*Legionella pneumophila*	1 locus	2 loci

Both the Sawyer’s run test and RDP show *L. pneumophila* has an intermediate rate of intragenic recombination when compared with other bacterial species.

Overall the collected evidence from this and several previous studies [12-14,16,17,23] strongly suggest that *L. pneumophila* is not a purely clonal organism but also undergoes significant recombination. The results presented here suggest that *L. pneumophila* retains evidence for a clonal vertical inheritance of genetic material whilst also demonstrating strong evidence of recombination by horizontal transfer of genetic loci. Although there was some evidence for recombination within the SBT genes, the frequency was low and this indicates that new alleles are most likely to be generated by point mutations rather than recombination. The signal from vertical inheritance of genetic material through clonal lineages is still evident when examining the genetic information contained from seven *L. pneumophila* loci. However it is also clear that recombination happens often enough so that it is a significant force in shaping the population structure. This does not alter the utility of SBT as a means to discriminate between isolates of *L. pneumophila*, particularly for outbreak investigation, since the results indicate that it is far from being a panmictic organism. Although we cannot infer a rate of recombination from this study, the relatively low frequency of recombination suggests that recombination would be unlikely to take place in the timescale of an outbreak and therefore the ST of isolates involved in an outbreak is also unlikely to change.

### Sequence Based Typing analysis: Clustering

**Figure 2:**
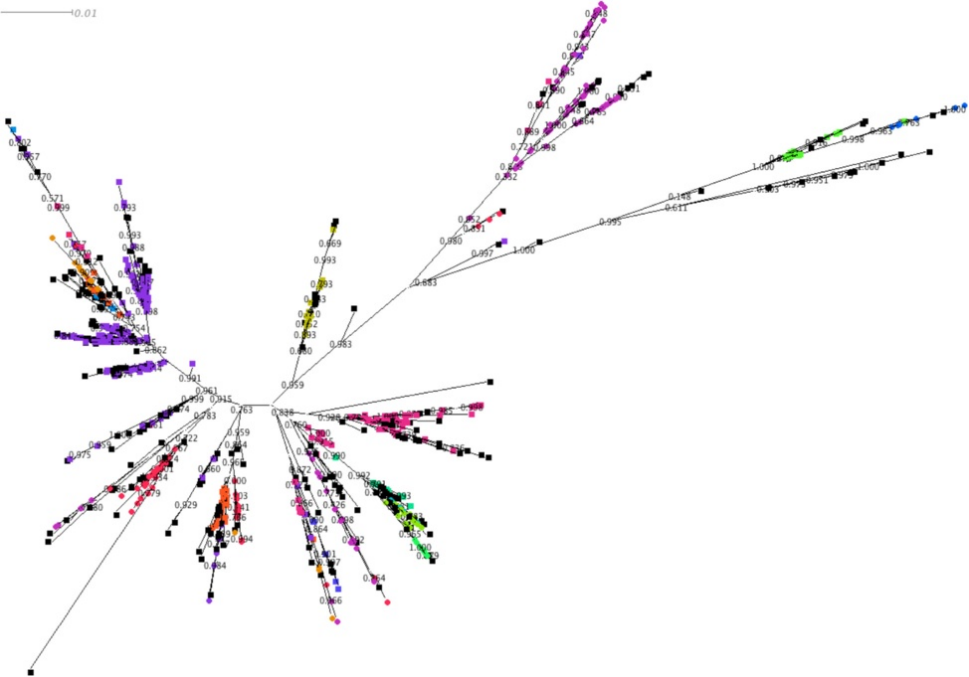
**Clusters as determined by eBURST mapped onto a radial phylogram generated by FastTree 2.** STs not assigned to a cluster (singletons in eBURST) are coloured black.

**Figure 3:**
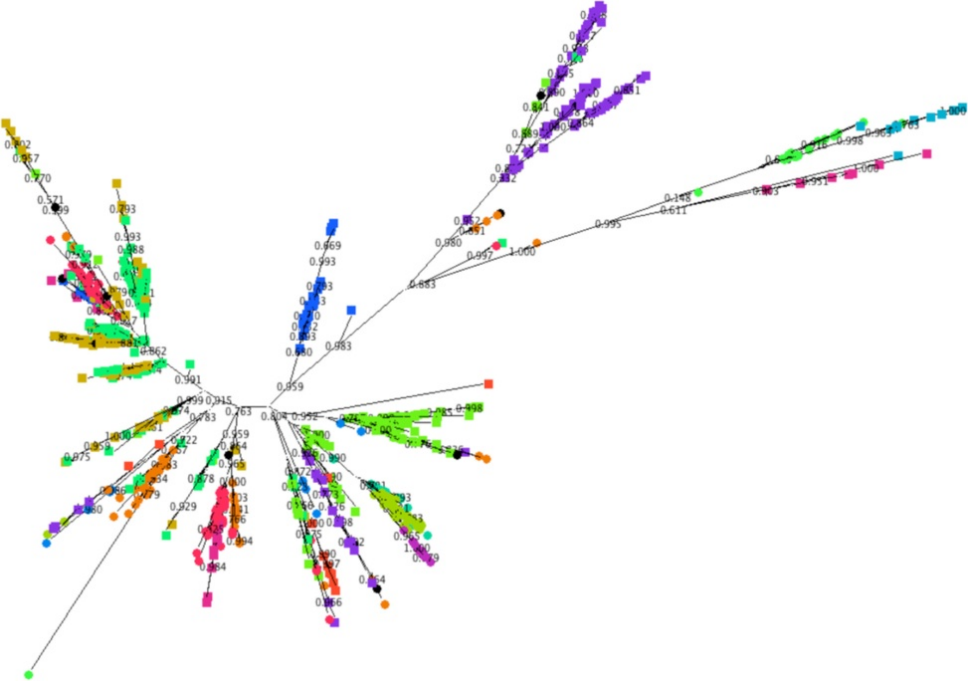
Clusters as determined by BAPS using allelic data mapped onto a radial phylogram generated by FastTree 2.

**Figure 4:**
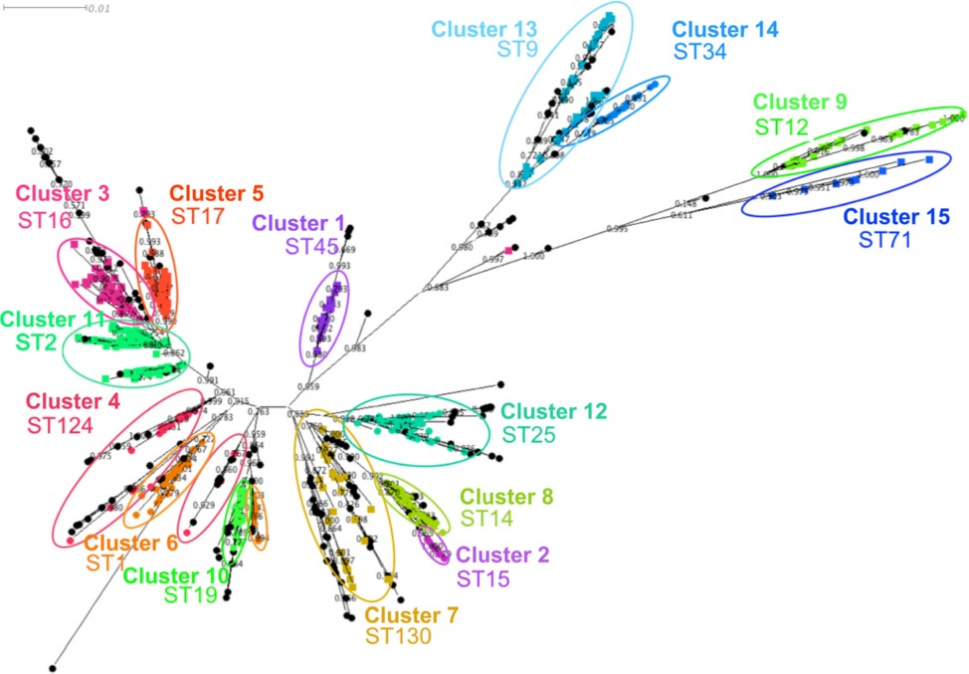
**Clusters as determined by BAPS using linked sequence mapped onto a radial phylogram generated by FastTree 2.** STs that have significant admixture are coloured black. The clusters are labelled using the lowest ST number found within the cluster.

Since the ultimate aim of this work was to find a practical way to cluster *L. pneumophila* isolates, a method of determining which clustering method resulted in the most accurate sub-groups was required. Given that the recombination analysis above indicates that clonal vertical inheritance plays a major role in the evolution of *L. pneumophila,* a phylogenetic tree based on the genetic distance between the concatenated sequences from the SBT loci will provide an approximate representation of the evolutionary history. Therefore a maximum likelihood (ML) tree was produced (data not shown) using RAxML [24], however bootstrapping proved computationally difficult given the sequence length and number of sequences. Therefore in order to obtain local support values for the branch split points the same data were used to produce an approximate ML tree with local support values using FastTree 2 [25]. This tree had almost identical topology to the RAxML tree and the majority of split points had local support values of > 0.8. The same sequence data used to generate the tree were clustered using three methodologies; eBurst, BAPS of allelic data and BAPS of sequence data (Figures 2, 3 and 4).

### eBurst analysis

eBurst uses the BURST algorithm to identify mutually exclusive groups of related genotypes in the population, to identify the founding genotype of each group and to predict the descent from the predicted founding genotype to the other genotypes in the group [26]. The algorithm assumes that each allele is equally related to all other alleles of the same locus and as such assumes that recombination is a frequent event. eBurst clustering produced 55 groups, 31 of which contained just two STs, and 190 singletons.

### Bayesian Analysis of Population Structure (BAPS)

BAPS is a tool for the detection and representation of recombination between populations [27]. The BAPS mixture model is derived using novel Bayesian predictive classification theory, applied to the population genetics context. A variety of different prior assumptions about the data can be utilized in BAPS to make inferences, however it does not require either a prior model of clonality versus recombination, or a pre-defined number of clusters. BAPS can be used to determine the population structure, to determine gene flow within a population, to determine the amount of admixture in an individual, and to divide the population into clusters [28,29]. The data required for BAPS population analysis can be in several formats.

The first analysis performed used allelelic data identical to that for the BURST analysis but saved in GENEPOP format. Those STs that had significant (p <0.05) admixture (genetic material from more than one genetic lineage) were not assigned to a cluster. With the maximum permissible number of clusters set at 20 clusters, the optimal partitioning of the 838 STs resolved them into 15 clusters with a mean number of STs of 55.9 and a standard deviation of 48.0. However 12 sequence types had significant admixture and were excluded from clusters.

BAPS analysis was also performed using molecular sequence data. Both cluster and admixture analyses were carried out. As previously, those STs that had significant (p <0.05) admixture were not assigned to a cluster. With the maximum clusters set at 20, the optimal partitioning of the sequence types was again found to be 15 clusters with a mean number of STs of 55.9 with a standard deviation of 31.0. However in this analysis, 181 sequence types had significant admixture and were thus excluded from clusters.

The assignment of sequence types to clusters as determined by the three methods was visualised by colouring the nodes (representing the individual STs) of a radial phylogram drawn by Dendroscope [30] according to the cluster the ST belongs to (Figures 2, 3 and 4).

By comparing different clustering methodologies we aimed to identify one that would best fit the type of population seen in the species. The data presented show that both vertical inheritance of mutation and HGT/recombination play significant roles in shaping the genetics of *L. pneumophila* thus an appropriate method to sub-divide the population must take both into account. It was therefore anticipated that clustering methods deriving distance between strains based on sequence identity and allowing for admixture would most accurately divide the population into clusters that reflect the true origin of the members of the cluster.

Based on the ML tree, clustering using BAPS linked sequence analysis demonstrates the most consistent mapping of clusters to the topology of the clades within the tree. On one hand this is not surprising since the BAPS analysis and ML tree both have the same input data (seven locus sequence data). However it does illustrate that clustering based on allelic data alone, and assuming linkage equilibrium, produces very different results from that when the sequence is taken into consideration: BAPS analysis using sequence data takes into account both the evolution of sequence and the flow of genetic information between populations. Therefore we consider BAPS to represent a reasonable compromise between clustering based on standard phylogenetic techniques that assume linear evolution of sequences by mutation and clustering using the BURST algorithm that assumes a freely recombining population. Based on the BAPS linked-sequence clustering 15 clusters formed the most likely partition.

### Genome Sequencing

To assess if this BAPS analysis and clustering of the ST data remained valid when whole-genome data were considered, a rational approach was used to select isolates representative of each of the 15 clusters. These were sequenced using high throughput sequencing technologies (Table 3). These genomes should give a good overview of the diversity in the pan-genome of the species. The mean depth of reads using the Illumina technology is reported in Table 3. In all cases the depth was above the figure of 25 that is generally recommended for both SNP calling and *de novo* assembly using Illumina data.

**Table 3:** **Details of *Legionella pneumophila *** **strains used in the study**

**ST sequenced**	**BAPS cluster**	**Represen-tative ST**	**Strain**	**Reason for selection**	**Strain significance**	**Sequence source/technology**	**Mean Illumina read coverage**	**ENA accession number or reference**
46	1	45	H093620212	closest to centroid	fairly common clinical strain - assumed to be virulent	NGS mate paired Illumina	169	ERR315646
74*	1	45	H053260229	second of cluster	fairly common clinical strain - assumed to be virulent	NGS 454		ERR315647
15*	2	15	Lens	already published	caused a big outbreak in France	GenBank (NC_006369.1)		[31]
84	2	15	H043940028	closest available to centroid	only one available from this cluster	NGS paired end Illumina	283	ERR315648
47	3	16	H063920004	internationally significant	in top six strains that cause disease	NGS 454, paired end Illumina and mate paired Illumina	paired end 211 mate paired 227	ERR315649
47	3	16	Lorraine	already published	in top six strains that cause disease	GenBank(NC_018139.1)		[23]
47	3	16	LP_617	already published	in top six strains that cause disease	EMBLBank(ERS166047)		[32]
54*	3	16	H065000139	closest to centroid	uncommon strain nothing known	NGS paired end Illumina	161	ERR315650
62	3	16	H064180002	internationally significant	in top six strains that cause disease	NGS 454		ERR315651
611	4	124	H090500162	only one in cluster	unique environmental isolate	NGS mate paired Illumina	276	ERR315652
87	5	17	LC6677	second of cluster	common serogroup 3 strain - does cause disease	NGS paired end Illumina	490	ERR315653
376	5	17	RR08000760	closest to centroid	unique environmental isolate	NGS mate paired Illumina	235	ERR315654
1*	6	1	Paris	already published		GenBank (NC_006368.1)		[31]
1	6	1	LP_423	already published		EMBLBank(ERS166048)		[32]
5	6	1	EUL00013 (83/41091)	on an interesting branch of ST001	only three in database - all from small outbreak in Glasgow	NGS mate paired Illumina	304	ERR315655
152	6	1	H074360702	closest to centroid	uncommon - mainly environmental	NGS mate paired Illumina	180	ERR315656
179	7	130	H093380153	closest to centroid	uncommon but causes disease	NGS paired end Illumina	32	ERR315657
337	7	130	RR08000517	second of cluster	uncommon strain appears to be phenotypically variable	NGS mate paired Illumina	161	ERR315658
42	8	14	130b (Wadsworth)	already published	in top six strains that cause disease - globally distributed. Isolated in USA in ~1980	GenBank (FR687201.1)		[33]
42	8	14	H044540088	internationally significant	as above but isolated in UK in 2004 - assumed to be virulent	NGS 454		ERR315659
44	8	14	H100260089	closest to centroid	similar to ST42 but not so common	NGS paired end Illumina	346	ERR315660
154*	9	12	LC677 4	closest to centroid	seen in Canada and UK as a cause of nosocomial LD	NGS mate paired Illumina	84	ERR315661
336*	9	12	Lansing-3 (sgp15TS)	second of cluster	Representative of *L. pneumophila* subsp *fraseri*	NGS paired end Illumina	150	ERR315662
23	10	19	H063280001	closest to centroid	in top six strains that cause disease	NGS paired end Illumina	265	ERR315663
78*	10	19	LC6451	second of cluster	unique strain - caused a major outbreak in Barrow in 2002.	NGS 454		ERR315664
51	11	2	Corby	already published/closest to centroid	uncommon but well characterised - virulent in animal model and protozoa	GenBank (NC_009494.2)		[34]
454	11	2	H091960011	second of cluster	unique environmental strain (from Roman baths in Bath)	NGS mate paired Illumina	268	ERR315665
578	11	2	Alcoy	already published	responsible for a very big outbreak in Spain	GenBank (NC_014125.1)		[35]
59	12	25	H070840415	closest to centroid	quite common environmental strain - a few cases of LD.	NGS mate paired Illumina	246	ERR315666
188	12	25	H075160080	second of cluster	no particular data	NGS paired end Illumina	298	ERR315667
36	13	9	Philadelphia	already published/closest to centroid	the type strain - well characterised caused the Philadelphia outbreak.	GenBank (NC_002942.5)		[36]
37	13	9	H034680035	internationally significant	in top six strains that cause disease	NGS 454		ERR315668
186	13	9	H044500045	second of cluster	unique clinical isolate	NGS paired end Illumina	375	ERR315669
34	14	34	RR08000134	closest available to centroid	no particular significance	NGS paired end Illumina	301	ERR315670
68	14	34	H074360710	second of cluster	no particular significance	NGS mate paired Illumina	179	ERR315671
707*	15	71	H091960009	only one in cluster	unique environmental strain (from Roman baths in Bath)	NGS paired end Illumina and mate paired Illumina	Paired end 136 Mate paired 50	ERR315672

Asterisks designate strains that are likely to have plasmids based on the analysis described in the methods section of this manuscript. The sequence data described can be obtained using the European Nucleotide Archive accession number listed in the table.

### *De novo* assembly

**Figure 5:**
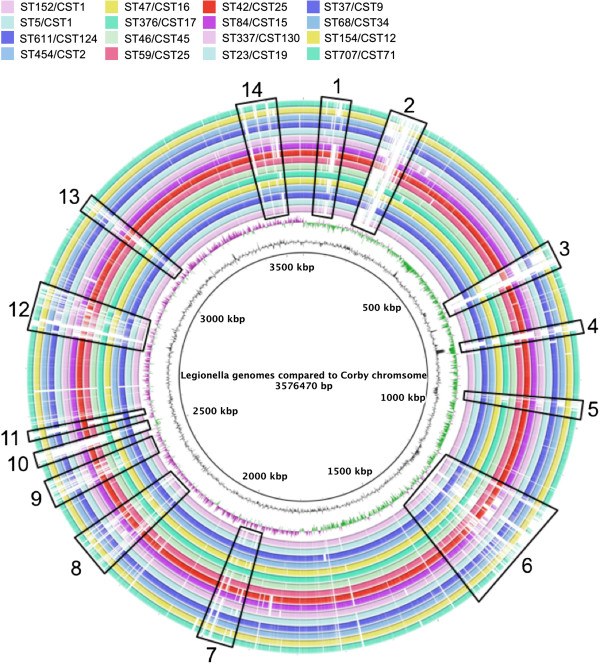
**BRIG blast analysis of the Legionella genomes using the genome of Corby as a reference.** The strains and figure colours used were from centre to outside ST152 (CST1 mauve), ST5 (CST1 light blue), ST611 (CST124 dark blue), ST454(CST2 medium blue), ST47(CST16 leaf green), ST376 (CST17 dark green), ST46(CST45 light green), ST59 (CST25 pink), ST42(CST14 red), ST84 (CST15 purple), ST337 (CST130 mauve), ST23 (CST19 light blue), ST37 (CST9 dark blue), ST68 (CST34 medium blue), ST154 (CST12 leaf green) and ST707 (CST71 dark green). Regions showing significant variability in genomic content are boxed with black lines and labelled 1–14.

The reads were assembled *de novo* into scaffolds. The genomic content of these scaffolds was assessed using BLAST Ring Image Generator [37] where the scaffolds were the query sequences and the reference sequence was the genome from the Corby strain (Figure 5). Corby was chosen since it is known to be virulent in both humans and animal models and has extra mobile genetic elements not seen to date in the other sequenced legionella genomes [34,38]. Regions showing a high level of variability compared to the Corby genome were investigated further by looking at the gene content of those regions (Additional file 1: Table S1).

The graphical output from the BRIG analysis comparing the genomes to the Corby sequence displays an overview of the major regions of variability among these genomes such that 14 regions of substantial variation were observed (Figure 5 and Additional file 1: Table S1). Many of the genes present in these regions are phage or transposable-element associated, suggesting that much of this variability is driven by mobile elements. Many of these regions are adjacent to or have a tRNA sequence within them, a common location for mobile element integration [39]. Several of the variable regions have genes involved in a conjugation/type IV secretion system (T4SS). The excision, transfer and re-integration of genetic loci by this class of genes has been implicated in HGT [34]. Variability in T4SS genes has been shown previously to be a major contributor to the genome plasticity of *L. pneumophila*[23]. Other classes of genes include those encoding transporter/eflux proteins, proteins involved in glycosylation, putative virulence proteins, restriction endonuclease system proteins, and antibiotic resistance proteins. None of these proteins are involved in core metabolic functions and variability in the presence and absence of these genes is likely to result in phenotypic changes that alter the ability of the organism to survive within its environment.

### Plasmid analysis

Apart from acquisition of genomic islands another common way that bacteria gain genetic elements that confer phenotypic differences is by plasmid acquisition. In order to investigate the presence of plasmids in the genomes the plasmids of the Lens and Paris genomes were compared. A shared 9.2 kb region was used to query both the assembled and GenBank genomes. Although there may be plasmids circulating in the population that do not contain this shared locus, the same sequence is also present in the plasmid of another *Legionella* species, *Legionella longbeachae* (NSW150 plasmid pLLO: Accession FN650141) suggesting that this is a conserved sequence present in at least some of plasmids of the *Legionella* genus. Blast analysis detected this conserved plasmid sequence in a small proportion of the strains (8/33) and the plasmids sequence itself was variable. The following genomes produced a hit whose e-score was less than 1x10^-20^: Lens: (100% identity over 9299 bases), Paris: (83% identity over 8319 bases), ST154: (83% identity over 7270 bases), ST336: (83% identity over 7270 bases), ST44: (88% identity over 249 bases), ST54: (99% identity over 9299 bases), ST707: (83% identity over 7373 bases), ST74: (82% identity over 8239 bases), ST78: (83% identity over 7323 bases). It can be seen that there are some closely related strains (ST 154 and 336 in the same cluster) that share a very similar plasmid whereas other closely related strains (e.g. Paris, ST5 and ST152) have different plasmid content. It is likely therefore that the plasmids are exchanged horizontally between strains and have a different heredity to the chromosome. It should be noted that since the sequencing in this project is only draft sequence it is not possible to derive the complete plasmid sequences and hence their content. It is probable that the small amount of matching sequence in the ST44 strain is not from a plasmid.

### Phylogeny based on gene content

**Figure 6:**
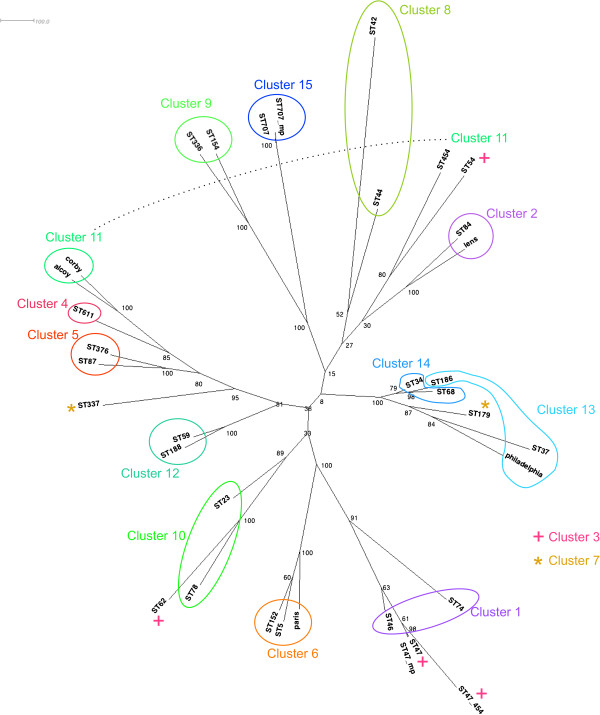
**A maximum parsimony tree based on the presence and absence of genes in the 27** ***L. pneumophila *genomes sequenced as part of this work and 5 additional genomes from GenBank (Alcoy, Corby, Lens, Paris, Philadelphia).** The internal nodes are labelled with the bootstrap values.

To assess variation among the genomes based on differences in gene content between the genomes, putative genes from all the genomes were grouped using cd-hit into clusters where each cluster member is homologous to one another. The clusters represent proteins shared between the genomes, and the presence of a member within these clusters for a particular strain represents the existence of the gene for this protein within the genome of that strain. There were 2173 clusters containing members from every strain sequenced (representing those genes found in all genomes) corresponding to, on average, 67.9% of the total number of genes in each genome. The mean percentage of genes shared between clusters was 85.8% (standard deviation 3.7%) and a range of 74.8% to 98.8%. The clusters were used to generate a matrix of 1 and 0 s corresponding to the presence or absence of a gene in each of the strains. This matrix was used as the input for a parsimony analysis, which generated a tree with the most parsimonious representation of the data (Figure 6).

### Phylogeny based on SNP variation

**Figure 7:**
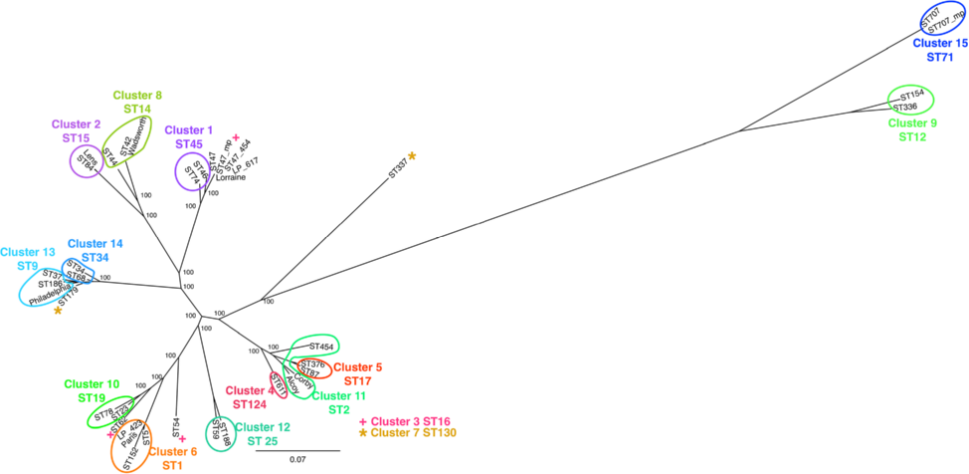
**A maximum likelihood tree based on the SNP differences between all 27** ***L. pneumophila *genomes sequenced as part of this work and 5 additional genomes from GenBank (Alcoy, Corby, Lens, Paris, Philadelphia).** Also included are four additional genomes from external sources (LP_423(ST1), Lorraine (ST47), LP_617 (ST47), Wadsworth (ST42)) used for intra ST-comparison. The internal nodes are labelled with the bootstrap values. The data for this tree can be viewed at http://purl.org/phylo/treebase/phylows/study/TB2:S15085.

**Figure 8:**
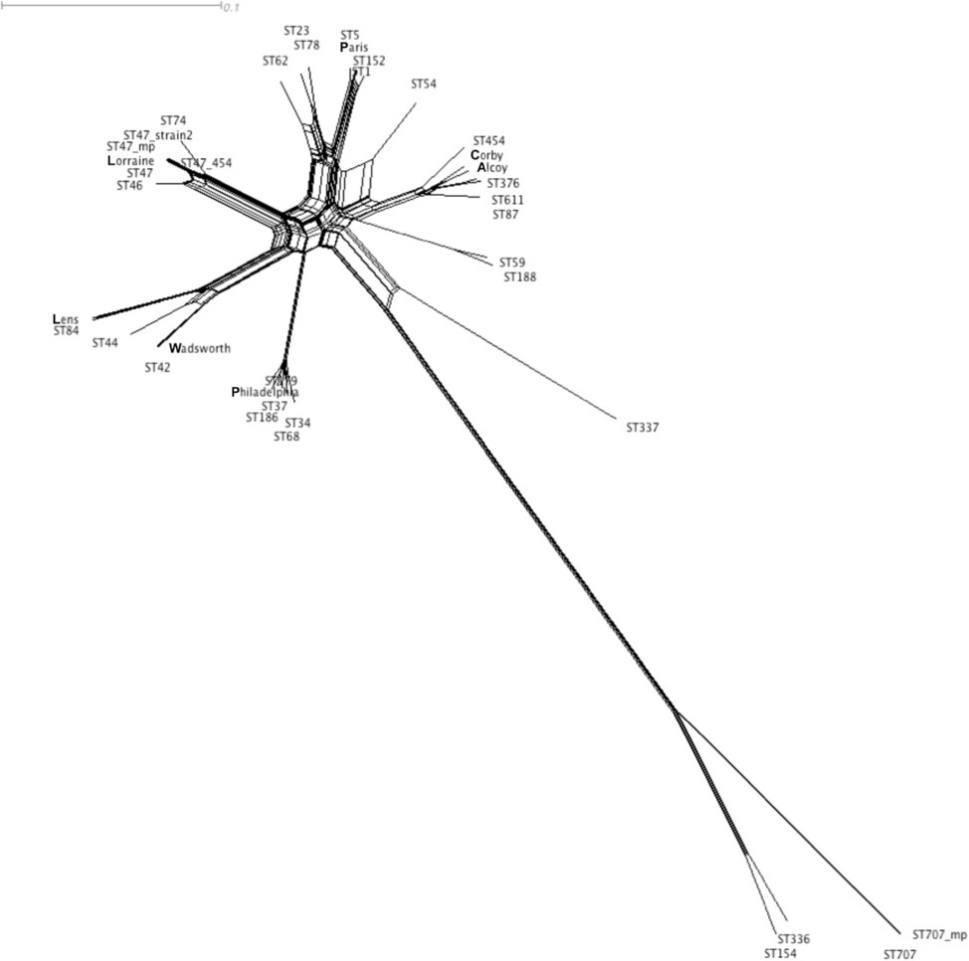
A reticulate tree generated by the Neighbor-net algorithm of SplitsTree4 using the concatenated SNPs from the genome sequences of 33 strains as input data.

An alternative way to assess variation among the genomes is to examine single base polymorphisms. To achieve this Illumina reads, or synthetic wgsim reads, were mapped to the Corby genome and high quality SNPs extracted for those positions conserved in all genomes. The nucleotides present in each strain at all SNP positions were concatenated and used to generate a maximum likelihood tree (Figure 7). The same SNP data was used as input for the Splits Tree program and a reticulate network tree was drawn using the Neighbor-net algorithm (Figure 8).

A number of observations are pertinent when examining these data. Two isolates (H063920004 and H091960009) were sequenced with different technologies. H063920004 with Illumina paired end, Illumina mate-paired and Roche 454, H091960009 with Illumina paired end and Illumina mate-paired. There were no SNP differences between the sequences of these replicate samples demonstrating that the protocol used for calling SNP variants is both robust and consistent. There were three isolates of ST47 (labelled ST47, LP-617 and Lorraine), two from the UK and one from France, each isolated in a different year between 2003 and 2006. These differed by just four SNPs. Two ST42 isolates, from the UK and USA (labelled ST42 and Wadsworth), were isolated 20 years apart and only exhibited 20 SNP differences. In contrast two ST1 isolates, a representative of the ‘Paris’ strain and a UK strain sequenced as part of another study, were isolated within 2 years of each other yet these exhibited 280 SNP differences.

These results show that lineages of *L. pneumophila* contain differing levels of observable diversity. There are several evolutionary scenarios that could be postulated as explanations for these observed differences. A lineage that occupies a niche where there is strong purifying selection will be less diverse. Conversely a lineage that is the result of rapid expansion within a previously unoccupied niche will tend to be more diverse. One likely scenario is that ST1 is a successful clonal lineage that emerged before the ST47 lineage and therefore has had more time to diversify by genetic drift. It is also possible that each lineage of *L. pneumophila* will be subject to differing selection pressures when infecting a human host, even though this is effectively an evolutionary dead-end. One possible scenario is that the majority of ST1 strains and a limited number of sub-lineages of ST47 cause disease in humans. If this is the case then a likely explanation is that the common ancestor of the ST1 lineage was able to infect the human species and the ancestor of the ST47 lineage did not replicate effectively in a human host. Subsequently a minority of descendents of the ST47 lineage have acquired the ability (through mutation, gene loss or acquisition) to cause human infection. Differentiating between these putative evolutionary scenarios will be difficult and will require a greater understanding of the effects of diversity within the lineages of *L. pneumophila* sampled from the environment and human infections.

When examining the output from the Splits Tree analysis, the more splits observed, the more recombination or HGT is likely to have taken place. The majority of clades in the tree show a branching network structure suggestive of frequent recombination. The Phi test for recombination as implemented in SplitsTree also showed evidence for recombination (p = 0.0). The exceptions are the clade(s) containing ST136/154 and ST707. The ST136/154 clusters contain members of the sub-species *L. pneumophila* subsp. *fraseri*. This would explain the long branch length for this cluster and the genetic diversity among these strains and the rest of the population could be responsible for the low levels of horizontal exchange and recombination with the remainder of the *L. pneumophila* strains.

The maximum likelihood tree based on SNPs and the maximum parsimony tree based on gene presence can be used to compare clustering based on whole genome data with that based on the SBT data. In both genome trees the strains making up the majority of clusters identified by BAPS analysis of the seven SBT loci group together. This is most evident in the tree resulting from the SNP analysis. This tree and its branch lengths is mostly likely to match the true evolutionary history of the strains since, for all but the most panmictic organisms, the well understood evolutionary mechanisms causing mutations in the genome will be summarised by the SNPs occurring in positions sampled across the genome. The selection of core SNPs (those SNPs in locations found in all genomes) for analysis obviates the problems associated with using SNPs that are in genes that are variably present in different genomes and in loci associated with transposable elements. Some of the SNPs will be in loci that have acquired by HGT/recombination and will not match the evolutionary history of the core genome. The reason for this is that a large number of SNPs, that would have taken considerable time to arise by the process of DNA mutation, can be introduced by a single HGT event. However since *L. pneumophila* only shows moderate recombination there should be enough ‘signal’ from the SNPs in loci that have not undergone HGT to mask the ‘noisy’ data arising from SNPs arising from HGT. In the tree derived from the presence of genes in the different genomes (Figure 6) there is more evidence for strains from BAPS clusters being split over more than one branch of the tree. This is likely to be due to the fact that HGT of genes can result in large changes in presence and absence data and this tree reflects the fluid nature of the *L. pneumophila* genome, especially the non- core genome. One reason that may explain differences between the SBT and genome-based trees is that several of the genes that make up the SBT scheme are possibly under positive selective pressure. These include genes encoding surface proteins (*fla*A, *momp*S and *pilE*) and factors that may be involved in virulence (*pro*A and *mip*) [3,4]. This is in contrast to the majority of genes in the genome which will be evolving neutrally. However although there are clear differences between the two trees, particularly in terms of the branch lengths, the overall topologies are broadly similar as measured by the groups of strains found within clades.

### Admixture analysis

In both trees strains from BAPS clusters 3 and 7 are split across sometimes quite distant branches of the tree. Re-examination of the data from the original BAPs analysis shows that these results may reflect admixture of the genetic material of some of the strains from these clusters. Admixture refers to the process by which two discrete populations exchange genetic material resulting in organisms that have a genome that is sourced from two different origins.

BAPS analysis will, for each sequence, estimate the proportion of genetic material arising from organisms from each of the clusters that are derived as part of the analysis. It will also assign a p value to the likelihood of an organism being admixed. The data shows that it is likely that strains belonging to STs 47, 54, and 179 have significant admixture and that there was not enough information in the seven loci to show this when performing the initial BAPs clustering.

This hypothesis was tested further by applying the same BAPS sequence-based clustering that was originally used to generate the clusters from 838 ST to a larger dataset which became available at the end of the study (1020 STs). These data are reported for the STs found in clusters 3 and 7 (Table 4). With the increased data available from 1020 STs the probability of these STs being admixed is now significant and it would not be possible to assign these STs to a cluster with statistical confidence. However for both ST62 and ST337 there is no significant admixture within either of the data sets and it is likely therefore that these are good representative strains for clusters 3 and 7 respectively.

**Table 4:** **Table showing admixture *of Legionella pneumophila *** **strains**

**Cluster**	**ST**	**Proportion of genetic material from clusters (838 strain data set)**	**Significant admixture? (838 strain data set)**	**Admixture analysis with 1020 strains**	**Significant admixture? (1020 strain data set)**
3	47	**3**: 0.77, **1**:0.21	no	**3**: 0.36, **1**:0.29, **11**: 0.35	yes
3	54	**3**: 1.0	no	**3**: 0.72, **10**:0.24	yes
3	62	**3**: 1.0	no	**3**: 0.97	no
7	179	**7**: 0.85, **13**:0.14	no	**7**: 0.56, **13**: 0.35	yes
7	337	**7**: 0.96	no	**7**: 1.0	no

The clusters listed are those that show aberrant clustering on both trees derived from whole genome data. Only those clusters (cluster numbers shown in bold text) that contribute more than 0.1 of the genetic material of a strain are reported.

In the original BAPS analysis STs 1, 5 and 152 were all assigned to cluster 6 with no significant admixture despite ST5 being in a separate clade on the phylogenetic tree derived from the seven locus sequence data. The prediction from this data was that whole genome data would show these strains to have similar ancestral origins. Both whole genome trees show this to be the case with all three STs clustering tightly in one branch of the tree.

## Conclusions

This paper describes the sequencing of multiple genomes from strains representing most of the diversity present in the *L. pneumophila* population sampled from both environmental sources associated with human habitation and from patients with Legionnaires’ disease. Based on genome comparison 2172 genes (approximately 70% of the mean number of genes per genome) are conserved in all strains which is less than that reported previously (approximately 2400 genes, which is equivalent to 80% of the mean number of genes per genome) [16,23]. This is probably due to the samples representing a wider breadth of the population than the genomes used to calculate the core genome size in previous studies. The remaining 30% of the genome, often known as the accessory genome, is composed of many classes of genes but common themes include those that encode for functions that can mobilise DNA and those that are involved in protein transport/secretion. The former may be responsible for driving a dynamic genome in the species by permitting many mechanisms for horizontal gene transfer. The latter could be involved with niche adaptation. This and other studies have shown that recombination is a significant driver of evolution of the *L. pneumophila* genome. However we show that the genetic signal contained in the seven loci of the SBT scheme is generally indicative of its genomic heritage. Some STs appear to have been derived from recombination between strains of two different genetic backgrounds. However by clustering STs using BAPs we can determine which STs are likely to exhibit admixture and therefore cannot be confidently assigned to a cluster. Future studies will include looking at strains within and between clusters to determine phenotypes that are shared within a cluster but differ between clusters, and subsequently to search for the genetic differences that correlate with these phenotypes.

## Methods

For *L. pneumophila* all STs up to and including ST850 (*n* = 838 after removing ‘withdrawn’ STs) were used in the study. A ST is ‘withdrawn’ when the depositor informs the database curators that the unique allelic profile was submitted in error and is in fact not extant. As comparator data the following MLST datasets (1 representative per ST) as present in the pubmlst.org data (July 2010 and downloaded from the links present at the URL http://pubmlst.org/data/) were included; *Staphylococcus aureus* (clonal), *Streptococcus pneumoniae* (intermediate) and *Neisseria meningitidis* (panmictic).

### Tests for recombination

To examine recombination within the *L. pneumophila*, *S. aureus*, *S. pneumoniae* and *N. meningitidis* populations the following types of events were tested for:

### Recombination between genes (intergenic)

Three methods were used to test for this

a. Standardised Index of Association as Implemented in Start 2 [40].

b. Recombination to mutation ratio (r/m) ratio as implemented by ClonalFrame (http://www.xavierdidelot.xtreemhost.com/clonalframe.htm, [41]). The exact method used was as described by Vos et al. [42]. Parameters -x 100000 -y 100000 -z 100 -M -m (where is the Watterson estimate for the scaled mutation rate theta). This is calculated as the number of segregating sites (i.e., the number of polymorphic sites as calculated by DNAsp http://www.ub.edu/dnasp/) divided by the (n-1)th harmonic number where n is the number of samples.

c. SplitTree analysis. The concatenated sequences from the SBT loci for all STs were used as input for the SplitTree program (version 4.12.3) and the Neighbor-net algorithm used to draw a tree. The phi test for recombination as implemented in this program was performed.

### Recombination within genes (intragenic)

Two approaches were taken

a. Running the recombination tests within the RDP3 suite [43]. A locus was considered to have undergone significant recombination if two or more of the tests in the RDP3 suite were positive.

b. Applying the Sawyer’s run test (Implemented in Start 2).

### Clustering algorithms

#### eBURST

eBURST was used to cluster strains using the default settings: grouping strains sharing alleles at ≥ 6 of the 7 loci with at least one other ST in each group. The number of re-samplings for bootstrapping was 1000 [26].

### Bayesian Analysis of Population Structure (BAPS)

This methodology is described in detail in the references [27-29]. Clustering of individuals was performed on allelic data from STs formatted in GENEPOP format. Ten runs were performed setting an upper limit of 20 clusters. Admixture analysis was performed using the following parameters: minimum population size considered 5, iterations 50, number of reference individuals simulated from each population 50, number of iterations for each reference individual 10. BAPS analysis was also carried out using the clustering of linked molecular data functionality. The sequence data were saved in Excel (Microsoft) format. The same parameters for clustering and admixture were used as for the allelic data.

### Whole genome sequencing

#### Strains

Strains used in the study were either sequenced by Next Generation Sequencing (NGS) technologies or available through GenBank (Table 3).

At the time of the study the EWGLI SBT database contained data from 4272 strains from 43 countries (date 09/06/2010). The authors’ strain collection of strains in the database comprises 1110 clinical and environmental isolates, representing 222 ST obtained from 33 countries around the world. Although 77% of these were obtained from UK many of these STs are found worldwide and thus selecting strains only from the authors’ collection is unlikely to introduce a significant geographical bias. Strains were selected from the authors’ collection to represent all 15 BAPS clusters derived from SBT sequence data (Figure 4). The ST that was nearest to a notional centroid of each cluster was calculated as described below. Where possible this ‘nearest to centroid’ ST was used as a representative of the cluster for sequencing purposes. In all but one case, at least one other strain with a different ST from the ‘centroid ST’ was sequenced for each cluster. Where possible these strains were selected because the ST is of public health significance. Details are given in Table 3.

### Centroid of cluster calculation

For each cluster the sequences from the seven loci that make up the SBT scheme were concatenated and a distance matrix constructed using the program dnadist from the PHYLIP suite. These distances were scaled to 2 dimensions using the multidimensional scaling function cmdscale in R [44] these dimensions being treated as x and y coordinates. The central coordinate in x and y space was calculated using the mean of all coordinates. The Euclidian distance of each strain in the cluster to the centroid was calculated by Pythagorean mathematics using the x and y coordinates from the multiple dimensional scaling calculations.

### Sequencing

Genomic DNA from pure bacterial cultures from each of the strains was sequenced using either 454 or Illumina technologies. The strains sequenced by 454 used the titanium chemistry in conjunction with 8 kb insert libraries. Those sequenced employing the Illumina technology used 50 bp read lengths in conjunction with either a paired end or mate-paired 3 kb insert library. Several strains were sequenced using both 454 and Illumina technologies (Table 3).

### Assembly

The 454 sequences were assembled using the Newbler software (version 2.5) from Roche. Default parameters were used for assembly and scaffolding. The Illumina reads were assembled using Velvet version 1.1.05 [45]. The process was optimised using the velvet optimizer script from the Victorian Bioinformatics Consortium ( https://github.com/Victorian-Bioinformatics-Consortium/VelvetOptimiser) with a kmer range of 33 to 47. The additional options -*shortMatePaired2 yes -ins_length2 2500 -ins_length2_sd 500* were specified for reads from the 3 kb mate pair libraries. Contigs were joined into scaffolds using the SSPACE tool [46].

### Mapping and SNP calling

In order to discover SNPs using a single method for Illumina reads, 454 reads or complete sequences from GenBank, short ‘Illumina-style’ reads were simulated from 454 assemblies and GenBank-derived genomes. This was achieved using the wgsim program from the Samtools package [47] with these parameters -*e 0 -r 0 -N 3000000 -d 250–1 50–2 50*. This resulted in two fastq files representing 3 million paired end reads of 50 bp with an insert size of 250 bp equivalent to the reads from the paired end libraries from the experimental Illumina sequences.

Simulated or experimental Illumina reads from all strains was mapped to the genome sequence of the Corby strain using bowtie 0.12.7 [48] using the *–m1* parameter to exclude reads that map in more than one place on the reference sequence and tend to cause false positives when calling SNPs. The Sequence Alignment Map from the Bowtie mapping was sorted and indexed using samtools to produce a Binary Alignment Map (BAM). Samtools mpileup was used to create a combined Variant Call Format (VCF) file using each of the BAM file. The VCF file was further parsed using a simple script to extract only SNP positions that were of the high quality in all of the genomes and write out these SNPs into a multiple FASTA format file. High quality SNPs were defined as having an overall SNP quality value of > = 90, at least one of the genomes must have a high quality (quality value > = 30) variant base (1/1 in the VCF file) at the SNP position, and the position must not contain SNPs reported as heterozygous (0/1 in the VCF file).

### Coverage

The coverage of reads mapped to a reference genome was assessed using BEDTools ( https://github.com/arq5x/bedtools2) and the genomeCoverageBed function.

### Plasmid analysis

A query sequence of 9299 bases, positions 3036 to 12334 from Lens plasmid pLPL (Accession: NC_006366) was used to search blast databases using blastall (blastn program) from NCBI.

### Overview of genome similarity

BRIG (BLAST Ring Image Generator) was used to produce an image to illustrate the similarity between the Corby genome and one sequence from each of the BAPS clusters (except for Clusters 1 and 2 where two sequences were included, one from each clade on the phylogenetic tree produced from SBT data). Similarity was determined using BLASTn.

### Gene content analysis

A novel method was used to cluster the genes from all the genomes in the study. This method we have termed CoreAccess is reported in full in a paper currently under preparation. Briefly, the protein sequences of all genes from the genomes were used as input for the program cd-hit [49]. These genes were either those already annotated in the sequence files of the GenBank genomes or those predicted using Glimmer3 [50] trained using the Corby sequence genes. The proteins were clustered using cd-hit using a hierarchical approach, first clustering at a high percentage cut-off and then stepwise lowering of the cut-off and clustering the clusters from the previous step. The final cut-off was 80%. This hierarchical approach overcomes errors that can arise in single step clustering as described on the cd-hit website (cd-hit.org). The hypothesis underlying this methodology is that the clusters contain homologous proteins from the different genomes and as such represent groups of proteins with the same or similar function from the different genomes. In order to be able to search the clusters and find for example genes shared by all the genomes, the information about the clusters in the cd-hit output was collated into a sqlite3 database using tools within the Core Access suite.

### Phylogenetic Tree construction

Maximum likelihood tree phylogenetic trees were produced from mutiple fasta files by the MEGA software package [51] using the Tamura-Nei model, and testing the phylogeny with 500 bootstrap replicates.

To construct a tree from the gene content analysis, the database generated by CoreAccess was queried using SQL so that the presence/absence of a protein representative from each strain in every cluster was recorded to produce a phylip compatible discrete state (binary 0/1) character matrix. The seqboot program for the Phylip package [52] was used to create 100 bootstrap replicates using the Discrete Morphology data type and Non-interleaved as parameters. Applying the Phylip pars program produced 100 trees from the bootstrapped data which were subsequently fed into sumtrees [53] to result in a final consensus tree including both branch length and bootstrap values. The parameters employed were --to-newick and --no-summary-metadata. Bootstrap values were converted to a percentage value using a custom BioRuby [54] script.

## Competing interests

The authors declare that they have no competing interests.

## Authors’ contributions

AU carried out the clustering plus whole genome sequence analysis and wrote the manuscript. GJ performed the recombination analysis and contributed to pilot clustering analyses. MM performed the laboratory work including DNA extraction and Sanger sequencing. NF coordinated the laboratory work and helped in the study design. TH conceived of the study, and participated in its overall design and coordination and helped to draft the manuscript. All authors read and approved the final manuscript.

## Supplementary Material

Additional file 1: Table S1 Table showing major regions of variability between the *Legionella* genomes as determined by blastn against the Corby genome. For each region some of the more notable features are listed. Click here for file
