# Supplementary material for: Comparison of the Legionella pneumophila population structure as determined by sequence-based typing and whole genome sequencing
Source: BMC Microbiol. 2013 Dec 24;13:302. doi: 10.1186/1471-2180-13-302 (PMC3877988; doi:10.1186/1471-2180-13-302)
Supplement: Additional file 1: Table S1 — Table showing major regions of variability between the Legionella genomes as determined by blastn against the Corby genome. For each region some of the more notable features are listed. [file 1471-2180-13-302-S1.doc]

| **Variable Region**  **Position and notes** | **Description of notable features within region** |
| --- | --- |
| **1**  62956-85510  Adjacent to tRNA-Asn | LPC_0065: Dot/Icm system substrate protein SdbB  LPC_0076: Multidrug resistance protein  LPC_0084: IS652 transposase  LPC_0085: Integrase |
| **2**  181809-322854 | LPC_0166: Phage repressor  LPC_0167: Vir region protein  LPC_0170 - LPC_0180: Conjugal transfer proteins Trb B-J and L  LPC_0184 - LPC_0191: Conjugal transfer proteins Tra C,D,F,G,I,J,L,M  LPC_0199: Lamdoid prophage RAC integrase  LPC_0219: Mrr restriction system protein  LPC_0233, LPC_0251: Transporter proteins  LPC_0259: chloramphenicol resistance protein  LPC_0271: catalase/(hydro)peroxidase KatG  LPC_0288, LPC_0290, LPC_0300: Transporter proteins  LPC_0302: multidrug efflux protein |
| **3**  609848-656813  Adjacent to tRNA-Pro | LPC_2822: GNAT family transporter acetyltransferase  LPC_2818: Site-specific recombinase  LPC_2815: Vir region potein  LPC_2812 - LPC_2802: Conjugal transfer proteins Trb B, D, E, F, G, H, I, J, L  LPC_2801 - LPC_2791: Conjugal transfer proteins Tra C,D,F,G,I, J, L, M  LPC_2790: Restriction enzyme M protein  LPC_2788: Restriction enzyme R proten  LPC_2785: RNA helicase  LPC_2782: Proline/betaine transporter  TP3 repeat |
| **4**  781146-800015 | LPC_2649: Hypothetical large (18.8kb) protein |
| **5**  942041-959170 | LPC_2530: O-antigen glycosyl transferase  LPC_2524: Spore coat poylsaccharide biosynthesis protein E  LPC_2520: LPS O-antigen ABC transporter wzm, wzt  LPC_2517: O-acetyl transferase |
| **6**  1176181-1351762 | LPC_2308: Aminoglycoside acetyltransferase  LPC_2307: Integrase  LPC_2298: GNAT family transporter acetyltransferase  LPC_2271: Cobalt/zinc/cadmium efflux protein  LPC_2262 - LPC_2260: Chemiosmotic efflux system B proteins A,B and C  LPC_2258: Metallo beta-lactamase transporter  LPC_2254: Copper efflux ATPase  LPC_2251 - LPC_2249: Chemiosmotic efflux system C proteins A,B and C  LPC_2243: Cation efflux protein  LPC_2214: Cold shock domain family transporter protein  LPC_2205: Phage-like AbiD protein  LPC_2204: Integrase  TP1 repeat  TP2 repeat  LPC_2189: IS30B/C/D transposase  LPC_2173: Virulence factor MVinN  LPC_2156: Integrase  LPC_2155: Transposase IS911  TP1 repeat  LPC_0573: TnpA transposase |
| **7**  1980717-2003564 | TP3 repeat  LPC_1145: 16KD immunogenic protein  TP2 repeat |
| **8**  2180061-2267908  Adjacent to two tRNA-Glu, tRNA-Ala and two tRNA-Phe | LPC_1352: Endonuclease  LPC_1384: TnpA transposase  LPC_1389: Putative ISS376 transposase  LPC_1395: Phage integrase  TP1 repeat |
| **9**  2413252-2461132  Adjacent to two tRNA-Lys and tRNA-Arg | LPC_1546: Cold shock DNA binding domain containing protein  LPC_1548: Cobalt/zinc/cadmium cation transporter  TP1 repeat  TP2 repeat  TP4 repeat  LPC_1563: Endonuclease  LPC_1564: ISSod6 transposase  LPC_1579: GGDEF domain containing sensory box protein |
| **10**  2493848-2532367 | LPC_1605: Dot/Icm system substrate protein sdeB  LPC_1606: Ankyrin repeat family transporter protein  LPC_1616: ISSod13 transposase |
| **11**  2556234-2587987 | LPC_1640: Sensory box histidine kinase/response regulator  LPC_1648: Drug efflux protein  LPC_1652: IcmL-like protein  TP1 repeat  TP2 repeat |
| **12**  2779608-2853021  Adjacent to tRNA-met | LPC_1843: Cadmium efflux ATPase  LPC_1847, LPC_1849: Cation efflux permease and outer membrane protein  LPC_1850: Reverse transcriptase  LPC_1856: Transposase IS4  LPC_1857: Phage repressor protein  LPC_1858: Vir region protein X3  LPC_1874: Type IV secretory protein virB4  LPC_1876: Pilus retraction ATPase  LPC_1880: Conjugative coupling factor TraD  LPC_2136: TnpA transposase  LPC_2132: TraK  LPC_2127: S652 transposase  LPC_2123: Integrase  TP1 repeat |
| **13**  3067186-3081617  Adjacent to tRNA-Pro | LPC_1891: Integrase  LPC_1889: TnpA transposase |
| **14**  3443118-3517672 | LPC_3180: ISSod6 transposase  LPC_3222: resolvase |

**Additionl file 1 Table S 1**: Table showing major regions of variability between the *Legionella* genomes as determined by blastn against the Corby genome. For each region some of the more notable features are listed.
